# Does concern regarding climate change impact subsequent mental health? A longitudinal analysis using data from the Avon Longitudinal Study of Parents and Children (ALSPAC)

**DOI:** 10.1098/rsos.251099

**Published:** 2025-08-06

**Authors:** Daniel Major-Smith, Isaac Halstead, Katie Major-Smith

**Affiliations:** ^1^ Centre for Academic Child Health, Population Health Sciences, Bristol Medical School, University of Bristol, Bristol, UK; ^2^ Department of the Study of Religion, Aarhus University, Aarhus, Central Denmark Region, Denmark; ^3^ Sustainability, Creativity and Innovation Research Group, Plymouth Marjon University, Plymouth, UK; ^4^ School of Psychology, University of Plymouth, Plymouth, UK

**Keywords:** ALSPAC, climate anxiety, climate concern, mental health, longitudinal, causal inference

## Abstract

Climate change is having a substantial—and increasingly severe—impact on our planet, affecting people’s health, security and livelihoods. As a consequence, the concept of ‘climate anxiety’ has recently been developed to characterize the psychological and emotional impact of concern over climate change. However, whether climate anxiety—or less extreme manifestations such as climate concern—impacts subsequent mental health is uncertain. Numerous studies have identified an association between climate anxiety and worse mental health, but as most of this research is cross-sectional it is impossible to infer the direction of causation (e.g. does climate anxiety cause broader mental health, or do broader mental health problems cause climate anxiety, or is there bidirectional causation?). In this paper, we used longitudinal data from young adults (aged approx. 30 years old) in the Avon Longitudinal Study of Parents and Children (ALSPAC) based in the UK. We first aimed to answer the following research question: does concern regarding climate change cause subsequent mental health? Our outcomes were a range of validated mental health scales for depression, anxiety and well-being, and analyses adjusted for a range of baseline confounders and prior mental health to try and estimate an unbiased causal effect. As a second research question, we explored whether the association between climate concern and mental health is moderated by whether participants engage in climate action and whether they believe that individual actions can mitigate the impacts of climate change. We found little evidence for a causal effect of climate concern on subsequent mental health or well-being, or for moderation of this relationship by these climate change beliefs and behaviours. Our results suggest that—in this population of young adults in the UK, at least—concerns regarding climate change do not, on average, appear to cause subsequent mental health issues. However, we stress that these results apply only to *climate concern*, and may not be generalizable to more extreme manifestations of *climate anxiety*.

## 1. Introduction

Climate change is increasingly affecting our planet, impacting people’s health, security and livelihood, as well as wider biodiversity [1]. At current rates of greenhouse gas emissions, our planet’s climate is expected to get more extreme and more volatile, increasingly affecting both people and the environment. The consequences of inactivity and ‘business-as-usual’ are therefore predicted to be dire, including increased displacement of people, famine, catastrophic weather events, biodiversity loss and the disappearance of countries and communities close to sea-level [1].

Given these devastating predictions—especially when coupled with the slow progress and frequent lack of political will to reach net-zero greenhouse gas emissions [2]—it is perhaps not surprising that many studies have identified ‘climate anxiety’ (or ‘eco-anxiety’) as an emotional response to these events, even in individuals not yet directly affected by climate change [3,4]. Although various definitions have been proposed [5], climate anxiety has been broadly defined as a ‘heightened emotional, mental or somatic distress in response to dangerous changes in the climate system’ [6]; or, perhaps more starkly, as ‘a chronic fear of environmental doom’ [7]. Climate anxiety has been associated with a range of symptoms including panic attacks, helplessness, anger, sadness, sleeplessness and irritability [5,6], and may particularly affect children and young adults [2,8].

Over the past 5 or so years, public awareness of climate anxiety has increased, with celebrities and high-profile names such as Greta Thunberg reporting to suffer from climate anxiety (e.g. [9–11]). Numerous studies have found that anxiety regarding climate change is associated with worse mental health, such as higher rates of depressive and anxiety symptoms [12–15], as summarized by Clayton [3] and Coffey *et al*. [5]. However, climate anxiety may not always be pathological, as it can also lead to positive and adaptive responses such as increased climate action [3]. This includes performing a greater number of pro-environmental behaviours and engaging in environmental activism [16]; such adaptive behavioural responses—and in particular collective, as opposed to individual, climate actions—have been suggested to mitigate the negative aspects of climate anxiety (e.g. [17]). Given these relationships, there have been a number of reports claiming that climate anxiety is a potential mental health crisis and offering advice to help minimize the impact of climate anxiety, by groups such as the Climate Psychology Alliance [6] and the American Psychological Association [7].

However, much of this work is cross-sectional (for a rare exception, see [18]) and on small and non-representative samples. This makes it difficult to know whether climate anxiety does in fact cause subsequent mental health, and—if so—how best to support those with climate anxiety (note that, following standard practice in causal inference literature [19], throughout this paper our use of ‘cause’ is agnostic regarding the direction of effect). For instance, it is plausible that prior mental health may cause both climate anxiety and subsequent mental health, meaning that longitudinal data are needed to adjust for prior mental health in order to remove this bias due to reverse causality and try to estimate an unbiased causal effect [20]; given this, there have been calls for longitudinal work to try and tease apart these complex causal relations [21]. Furthermore, the lack of large-scale population-based studies with representative samples may limit the generalizability of previous results, be unable to detect relatively small effect sizes, and potentially be biased due to selection (e.g. if participants with an interest or belief in climate change were more likely to take part [19,22]). There is therefore a need to explore these questions using data from a large-scale longitudinal population-based study; this is what we intend to do here, using data from the Avon Longitudinal Study of Parents and Children (ALSPAC) based in the UK.

Note that throughout the rest of this paper we predominantly focus on concern regarding climate change (‘climate concern’) rather than ‘climate anxiety’ specifically. As we discuss in more detail below, while climate concern and climate anxiety are correlated they are not synonymous, with climate concern reflecting the less-severe manifestations of climate anxiety [23]. However, we focus on climate concern here for several reasons. First, climate concern is much more common than climate anxiety in the population, with approximately 60% of 16−25-year-olds worldwide ‘extremely’ or ‘very’ worried about climate change [2]; for similar results, see [3,24]. Understanding the causal relationships between climate concern and mental health/well-being could therefore have important public health implications beyond focusing on more extreme manifestations of climate anxiety. Second, it is possible that the concept of climate anxiety—often characterized by clinical symptoms of depression and anxiety disorder (e.g. panic attacks, helplessness, sadness, sleeplessness [5,6])—conceptually overlaps with our mental health outcomes, and hence measure the same (or very similar) constructs. While not all scales of climate anxiety focus on these clinically relevant symptoms (e.g. [12]), we side-step this potential complication in our study by focussing on climate concern, rather than climate anxiety. Finally, from a practical perspective, we are also constrained by our secondary dataset, which only contains information on climate concern, not climate anxiety.

**Figure 1.**
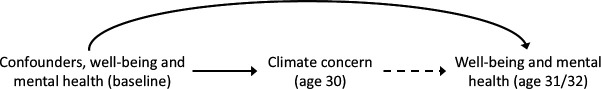
Directed acyclic graph (DAG) showing the assumed relations between climate concern, well-being/mental health variables and confounding variables in the present study. Arrows represent the direction of causality. Our causal effect of interest is the dashed arrow between ‘Climate concern (age 30)’ and ‘Well-being and mental health (age 31/32)’. Given the assumptions embedded in this DAG, even if climate concern does not cause subsequent mental health, in cross-sectional research we would still expect to observe an association between these variables due to confounding between the baseline variables (confounders and/or prior well-being/mental health) and both the exposure (climate concern) and the outcome (later well-being and mental health). By using longitudinal data, we can control for both baseline confounders and prior well-being/mental health, allowing us to close this back-door path between the exposure and outcome, and estimate an unbiased causal effect of interest (assuming the assumptions embedded in the DAG are correct). Note that baseline confounders (e.g. sex, socioeconomic position, etc.) and baseline well-being/mental health variables have been grouped together here for ease of presentation.

Our primary aim is therefore to use ALSPAC’s longitudinal data to explore whether climate concern may cause later mental health, adjusting for prior mental health status and a range of baseline confounders to rule out both confounding bias and reverse causality (figure 1). As a secondary aim, we will investigate whether this association is moderated by engagement in individual climate actions and/or belief that individual actions can impact climate change (i.e. whether participants who are concerned about climate change but do little to act on this, or who do not believe that their actions will have any impact, have worse mental health outcomes [17]). Our research questions are therefore as follows:

(1) Does concern regarding climate change cause subsequent mental health?
(2) Does engaging in climate action, or belief that individual climate action is effective, moderate the relationship between climate concern and mental health?

Note that as our aim is causal effect estimation rather than statistical hypothesis testing, these are phrased as broad research questions rather than specific hypotheses (see [25]).

## 2. Methods

This paper is a Registered Report, and the Stage I Report can be found at https://osf.io/8mbhs. The research questions, methods and analyses detailed below are identical to those specified in the Stage I Report, with only superficial updates and corrections (see electronic supplementary material, section S1 for full details).

This Registered Report was submitted to *Royal Society Open Science* following peer review and recommendation for Stage 2 acceptance at the Peer Community In (PCI) Registered Reports platform. Full details of the peer review and recommendation of the paper at PCI Registered Reports may be found at the links below. After submission to the journal, the paper received no additional external peer review, but was accepted on the basis of the Editor’s recommendation according to our PCI Registered Reports' policy: https://royalsocietypublishing.org/rsos/registered-reports#PCIRR.

Stage 1 recommendation and review history: https://rr.peercommunityin.org/articles/rec?id=793

Stage 2 recommendation and review history: https://doi.org/10.24072/pci.rr.100976

### 2.1. Study description

The current research focuses on the ALSPAC offspring generation. Pregnant women resident in Avon, UK with expected dates of delivery between 1 April 1991 and 31 December 1992 were invited to take part in the study. The initial number of pregnancies enrolled was 14 541, of which there were a total of 14 676 foetuses, resulting in 14 062 live births and 13 988 children who were alive at 1 year of age [26,27]. When the oldest children were approximately 7 years of age, an attempt was made to bolster the initial sample with eligible cases who had failed to join the study originally, resulting in an additional 913 children being enrolled. The total sample size for analyses using any data collected after the age of seven is therefore 15 447 pregnancies, resulting in 15 658 foetuses, of which 14 901 were alive at 1 year of age [28].

The final sample size consisted of all eligible offspring alive at 1 year of age who had not withdrawn consent for their data to be used, and who had data for either the exposure (climate concern) or any of the outcomes (mental health and well-being); the final sample size was 5146 participants. Note that combined we have extensive experience with ALSPAC data, including on climate change and mental health topics (e.g. [24,29–32]); however, prior to this Registered Report we had not analysed ALSPAC data for the specific research questions above as we had not yet accessed the mental health and well-being outcome data, and therefore did not know in advance the results of the proposed analyses.

Please note that the study website contains details of all the data that are available through a fully searchable data dictionary and variable search tool: http://www.bristol.ac.uk/alspac/researchers/our-data/. Study data gathered since the study offspring were aged 22 were collected and managed using REDCap electronic data capture tools hosted at the University of Bristol [33].

### 2.2. Data

#### 2.2.1. Climate concern exposure

Our main exposure of interest to indicate climate concern was the question ‘How concerned are you about the impact of climate change?’, with answers ‘Not at all concerned’ (2% of participants), ‘Not very concerned’ (9% of participants), ‘Somewhat concerned’ (48% of participants) and ‘Very concerned’ (41% of participants). As relatively few participants answered ‘Not at all concerned’, to boost sample sizes and power for the analyses in this paper we combined these answers with ‘Not very concerned’, resulting in a three-level categorical variable. A small number of participants (<50) did not answer this climate concern question because they answered that they ‘did not believe in climate change’ to a previous question; as individuals who do not believe in climate change presumably cannot be concerned about climate change, we coded these individuals as ‘Not at all concerned’. This question was asked as part of a questionnaire containing a larger section on ‘climate change’ between November 2021 and May 2022 when the study offspring were approximately 30 years of age [24].

While previous studies have used similar measures to assess climate anxiety and concern [2,16,18,34], we note that this measure of ‘climate concern’ may not fully capture all aspects of ‘climate anxiety’, especially when compared against validated climate anxiety scales (e.g. the ‘climate change anxiety scale’ [12]). Although similar measures of climate concern are moderately-to-strongly correlated with validated climate anxiety scales [23,35], it is possible for individuals to be highly concerned about climate change yet not anxious (although logically the inverse—low climate concern but high climate anxiety—would appear impossible). Nonetheless, previous studies investigating climate anxiety have used similar questions regarding climate concern or worry to explore climate anxiety [18], with these responses having high internal validity with related questions such as feeling ‘tense’, ‘anxious’ and ‘terrified’ regarding climate change [16]. While our measure of climate concern is not synonymous with climate anxiety, there is conceptual overlap, with recent research confirming that climate concern appears to capture the less-severe end of the climate anxiety spectrum [23].

#### 2.2.2. Individual climate actions and efficacy effect modifiers

We also used two further variables from this ‘climate change’ questionnaire as effect modifiers in follow-up analyses: engagement in individual climate actions and belief that individual actions can impact climate change. For engagement in individual climate actions, participants were given a list of 17 pro-environmental actions (including ‘reduced air travel’, ‘eaten less/no meat and/or dairy’ and ‘reduced household waste’; see electronic supplementary material, table S1 for a full list), and for each action asked whether they had ‘Not done this’, ‘Done due to climate change’ or ‘Done for other reasons’. The total number of actions performed for climate change reasons (max = 17; mean = 5.2) was used as our measure of engagement in climate action. For belief that individual actions can impact climate change, we used the question ‘Do you think that what you do, however small, will make a difference to the long-term effects of changes to our climate?’. The original response options to this question were ‘No’ (21%), ‘Not sure’ (27%) and ‘Yes’ (52%); for this study, to minimize the number of interaction terms and boost power we combined the ‘No’ and ‘Not sure’ responses to create a binary variable with the levels ‘No/Not sure’ and ‘Yes’.

#### 2.2.3. Mental health and well-being outcomes

We used a range of psychometrically validated well-being and mental health outcomes assessed after the climate questions. This included (scales summarized in electronic supplementary material, table S2):

(i) The 10-item Edinburgh Postnatal Depression Scale (EPDS) to assess depressive symptoms (note that this scale is valid for measuring depression more generally, as well as during the postnatal period [36]). Total scores range from 0 to 30, with higher scores indicating more severe depressive symptomatology. In the ALSPAC mothers, the EPDS had high internal validity (Cronbach’s alpha > 0.80) and construct validity when compared with other validated depression scales, such as the Centre for Epidemiologic Studies Depression Scale [37]. This EPDS was asked between June 2023 and January 2024, when the study offspring were approximately 32 years of age.
(ii) The 7-item Generalized Anxiety Disorder-7 (GAD7) scale to assess anxiety [38]. Total scores range from 0 to 21, with higher scores indicating more severe anxiety symptoms. Internal consistency of the GAD7 is high (Cronbach’s alpha = 0.92) and demonstrated construct validity when compared against other anxiety scales and clinical diagnoses of anxiety [38]. The GAD7 was asked at the same time as the EPDS, above.
(iii) The 14-item Warwick–Edinburgh Mental Well-Being Scale (WEMWBS) to assess well-being [39]. Total scores range from 14 to 70, with higher scores indicating greater well-being. The scale has high internal validity in population samples (Cronbach’s alpha = 0.91) and construct validity when compared against other well-being scales [39]. This WEMWBS was asked between December 2022 and May 2023, when the study offspring were approximately 31 years of age.

#### 2.2.4. Confounders

We adjusted for a range of confounders which, based on plausible assumptions and/or previous literature, may cause both the exposure (climate concern) and the outcomes (well-being, depression and anxiety). These confounders are summarized in electronic supplementary material, table S3, and include: prior well-being and mental health measures, offspring sex, ethnicity, relationship status, having children, various measures of socioeconomic position (e.g. highest education level, area-level deprivation quintiles and income), personality traits (here we focus on ‘openness to experience’, as it is associated with both climate beliefs and mental health in ALSPAC, and ‘neuroticism’, given its known associations with mental health [29,40]), as well as parental measures of depression, anxiety and socioeconomic position [34,41–45]. Note that we have not included ‘age’ here, as all study offspring were approximately the same age, meaning that age is unlikely to be a confounder.

All confounders were measured prior to the exposure and outcome, with most measured in early adulthood (between 21 and 28 years of age), other than personality assessed at age 13 and parental variables assessed during the pregnancy of the study offspring or shortly afterwards (electronic supplementary material, table S3). If the assumptions in figure 1 are met, adjusting for these confounders should result in an unbiased causal estimate of the exposure–outcome relationship [19]. However, whether these assumptions are met is impossible to verify empirically, and we will discuss sources of bias which may limit a causal interpretation—such as unmeasured confounding, selection bias and measurement error—in more detail in §4.

### 2.3. Analysis

#### 2.3.1. Research question 1—main effect of climate concern

We investigated the relationship between our climate concern exposure with each of the outcomes in turn (depressive symptoms, anxiety symptoms and well-being) using linear regression models. Analyses were repeated first in unadjusted (univariable) models, followed by adjusted (multivariable) models adjusting for all confounders detailed above. As ‘not at all/not very concerned’ will be the baseline exposure level for analyses, post-estimation tests were also used to assess whether the association with mental health outcomes differ between ‘somewhat concerned’ and ‘very concerned’ levels of the exposure. Sensitivity analysis to assess the levels of unmeasured confounding necessary to alter the study’s conclusions (e.g. making a result null, no longer reaching a given alpha level threshold, or how results would change if the level of unmeasured confounding was the same as the level of measured confounding) were to be applied, if relationships were found [46].

#### 2.3.2. Research question 2—effect modification

To explore whether the above main effects are moderated by engagement in climate actions and/or belief in individual efforts, we repeated the above analyses, this time including an interaction between climate concern with either ‘total number of individual climate actions’ or ‘belief in efficacy of individual actions’.

#### 2.3.3. Missing data

As is common with longitudinal population-based studies, as data were collected in multiple waves there are missing data in many of our variables. When focusing on our analytic sample (i.e. the 5146 participants with exposure or outcome data) levels of missing data in each variable are relatively small (mean missingness = 23%; min = 0%; max = 47%; see electronic supplementary material, table S4). Despite the low amount of missing data in each variable, as there are lots of confounding variables this resulted in a large degree of missingness in the final complete-case analysis; of the 5146 participants in the analytic sample, only approximately 420 (8%) had complete data on all variables.

Due to the large amount of missing data, complete-case analyses are likely to be inefficient (i.e. wider standard errors/confidence intervals). Missing data can also result in selection bias if missingness is related to the outcome [19,22,47]. However, as we adjusted for a range of variables known to relate to continued ALSPAC participation—such as maternal age, socioeconomic position, prior mental health and offspring sex [27,48,49]—this should minimize the extent of selection bias and we would expect complete-case analyses to be relatively unbiased [50]. Despite the inclusion of these predictors of selection, we cannot rule out the risk of selection bias, as we discuss in more detail below.

Although we expected the complete-case analyses to be largely unbiased, we imputed missing data via multiple imputation using chained equations to boost sample size and increase power [51,52]. The scenario described here—small amounts of missing data in a large number of variables—is ideally suited for multiple imputation as observed data from the other variables can be used to inform missing data in the other variables. We imputed up to the 5146 participants in the analytic sample, and used these multiply-imputed results as our main analyses. We also compared these multiply-imputed results against the complete-case results; as detailed above, we do not expect these complete-case results to be less biased than those from multiple imputation, but do expect multiple imputation to increase efficiency. For all multiple imputation analyses, we generated 50 imputed datasets with a burn-in of 10 iterations (this was checked to ensure convergence), with the imputation model specific to the variable of interest (e.g. logistic regression for binary variables, linear regression or predictive-mean matching for continuous variables, etc.).

For research question 1—the main effect of climate anxiety on mental health and well-being—we performed multiple imputation including all outcomes in the same imputation model. All of the exposure, outcome and confounder variables described above were included in this imputation model.

For research question 2 exploring potential effect modification, as there are additional complexities when imputing data with interactions to ensure that the imputation model is compatible with the substantive analysis model [52–54], we performed multiple imputation separately for these analyses. To simplify the process of including interaction terms in our imputation models, we also conducted multiple imputation separately for each outcome–effect modifier combination (rather than including all possible interaction terms in the imputation model). We first performed imputations using the ‘all interactions’ approach, which is necessary to ensure the imputation model is compatible with the analysis model and returns unbiased estimates (assuming the ‘missing-at-random’ assumption is met, which we assume it is here [54]). That is, when imputing the mental health or well-being outcome we included the exposure–effect modifier interaction in the imputation model; when imputing the climate anxiety exposure we included the outcome–effect modifier interaction in the imputation model; and when imputing the effect modifier we included the outcome–exposure interaction in the imputation model. Because best practice for the inclusion of interaction terms in multiple imputation is still a young and evolving field, we also included an additional multiple imputation method for interactions as a sensitivity analysis, known as multiple imputation by ‘substantive model compatible fully conditional specification’ (SMCFCS [53]). This approach is similar to standard multiple imputation, but uses rejection sampling to ensure that the results of the imputation models are compatible with the substantive analysis model. As with the multiple imputation analysis for research question 1, for both approaches all other covariates in addition to the exposure, outcome and effect modifier were included in all imputation models. If both the ‘all interactions’ and SMCFCS approaches provide similar answers, this will increase confidence that our results are robust. We also estimated the main effect of the exposure on the outcome to check that different imputation models provide similar results to those of the research question 1 analysis above.

All analyses were conducted in R version 4.3.1 [55], with standard multiple imputation and the ‘all interactions’ approach performed using the ‘mice’ package [51], SMCFCS performed using the ‘smcfcs’ package [53], and unmeasured confounding sensitivity analyses performed using the ‘sensemakr’ package [46]. As noted above, as our study focuses on causal effect estimation rather than hypothesis testing, the main focus of our results is on the range of plausible effect sizes (i.e. point estimates and 95% confidence intervals); *p*-values (interpreted as continuous measures of evidence against—on incompatibility with—the null hypothesis of no association) are interpreted alongside these effect estimates, in addition to *R^2^* statistics and predicted values/marginal effects from these models, to help interpret and contextualize results [56].

#### 2.3.4. Power analyses

Given the complexities of the dataset and analyses—many confounding variables, variables with missing data, the use of multiple imputation methods and uncertainty regarding the causes of missingness—all of which impact power, it is difficult to estimate an accurate minimum effect size this study would be capable of detecting. Nonetheless, we conducted a relatively simple simulation-based power analysis to estimate our power to detect a range of plausible minimum effect sizes for our research question 1 analysis (whether climate concern causes subsequent well-being and mental health), based on an expected complete-case sample of 1000 participants (note that this was our estimated complete-case sample size from the Stage I Registered Report, prior to accessing the data). For the purposes of this power analysis, we use an alpha level of 0.05, based on 1000 simulated datasets (see the ‘ClimateConcernAndMH_PowerAnalysis.r’ script [57]). Our plausible minimum effect size estimates were based on a range of effect sizes for a per-standard deviation (SD) increase in the mental health outcome, using the same effect size for both levels of the exposure (‘somewhat concerned’ and ‘very concerned’), with ‘not at all/not very concerned’ as the baseline; the effect sizes explored were 0.1, 0.2 and 0.3 (followed by 0.25) SD unit differences. As these effect sizes are on the standardized mean difference scale, they are comparable with Cohen’s *d* effect sizes.

Based on this power analysis using plausible parameter values, there was little power to reliably detect effect sizes of 0.1 SD difference (power for ‘somewhat concerned’ = 0.192; power for ‘very concerned’ = 0.176), or of 0.2 SD difference (power for ‘somewhat concerned’ = 0.603; power for ‘very concerned’ = 0.545). There was sufficient power to reliably detect an effect size of 0.3 SD difference over 90% of the time (power for ‘somewhat concerned’ = 0.939; power for ‘very concerned’ = 0.901), with moderate power to detect an effect size of 0.25 SD difference (power for ‘somewhat concerned’ = 0.798; power for ‘very concerned’ = 0.749). Given these assumptions, this suggests that our analyses for research question 1 likely have sufficient power to detect effect sizes of 0.25 SD unit differences or greater, and definitely above 0.3. However, given the complications mentioned above, this estimate may not be wholly accurate; for instance, if the confounders explain more or less variability in the outcome than our simulations assume then power may be either higher or lower, while using multiple imputation to boost the sample size may improve power (although it is unclear by how much as this will depend on the amount and patterning of missing data and how accurately the imputation models impute missing data values). Nonetheless, the results of this power analysis provide a useful benchmark regarding the minimum effect size of interest we can expect to reliably observe.

We also conducted power analyses for our research question 2 analyses, regarding potential effect modification of the above associations by both engagement in climate action (continuous variable) and belief in the efficacy of climate change efforts (binary variable). For engagement in climate action, our power analyses indicated moderate power to detect an interaction effect when a one-unit increase in climate actions was associated with a 0.05 SD improvement in mental health scores among those concerned about the climate (power for ‘somewhat concerned’ interaction = 0.794; power for ‘very concerned’ interaction = 0.692); to help contextualize this, we simulated climate action to have a standard deviation of 2, so a 2 SD increase in climate action would lower mental health scores by approximately 0.2 of a SD among individuals concerned about climate change. Power was substantially weaker when the interaction effects were set to 0.025 SD units (power for ‘somewhat concerned’ interaction = 0.291; power for ‘very concerned’ interaction = 0.242). For the power analyses with ‘climate efficacy’ as the effect measure modifier, there was sufficient power to detect an effect if belief in climate efficacy lowered mental health scores by 0.2 SD units among those concerned about the climate (power for ‘somewhat concerned’ interaction = 0.827; power for ‘very concerned’ interaction = 0.714), but not if the interaction effect size was 0.1 (power for ‘somewhat concerned’ interaction = 0.313; power for ‘very concerned’ interaction = 0.256).

Table S5 of the electronic supplementary material details the study design template summarizing the proposed study, which was included as part of the Stage I Registered Report.

## 3. Results

### 3.1. Descriptive statistics

Of the 4246 participants with data on climate concern (18% missing), 12% responded ‘Not at all/not very concerned’, 48% were ‘somewhat concerned’ and 40% were ‘very concerned’, as expected given our previous experience with these data. The ‘total number of individual climate actions’ (mean = 5.2; min = 0; max = 17; electronic supplementary material, figure S1) and beliefs regarding the efficacy of climate action (‘No/Not sure’ = 48%; ‘Yes’ = 52%) were also as expected.

Approximately 75% of the 5146 participants in the analytic sample had mental health and well-being outcome data. The mean scores and SDs were as follows: EPDS/depression (mean = 8.4; SD = 6.1 (electronic supplementary material, figure S2)); GAD7/anxiety (mean = 5.7; SD = 5.2 (electronic supplementary material, figure S3)); WEMWBS/well-being (mean = 46.8; SD = 8.9 (electronic supplementary material, figure S4)). The Cronbach’s alphas for all outcomes indicated ‘excellent’ internal reliability (EPDS = 0.90; GAD7 = 0.93; WEMWBS = 0.93). Descriptive statistics for the confounders are presented in electronic supplementary material, table S6.

Based on the raw data (table 1), there appeared to be little relationship between ratings of climate concern and any of the mental health outcomes. For depression and anxiety, participants who were ‘somewhat concerned’ about climate change had marginally lower scores than ‘not at all/not very concerned’, with ‘very concerned’ participants having slightly higher depression and anxiety scores. For well-being, ‘somewhat concerned’ and ‘very concerned’ had similar scores, which were both somewhat higher—indicating *better* well-being—than ‘not at all/not very concerned’.

**Table 1.** Summary of mental health and well-being outcomes by each level of the climate concern exposure (*n* for EPDS = 3258; *n* for GAD7 = 3245; *n* for WEMWBS = 3252). EPDS = Edinburgh Postnatal Depression Scale; GAD7 = Generalized Anxiety Disorder 7 scale; WEMWBS = Warwick–Edinburgh Mental Well-Being Scale.

climate concern	EPDS (depression)		GAD7 (anxiety)		WEMWBS (well-being)	
	*n*	mean (SD)	*n*	mean (SD)	*n*	mean (SD)
not at all/not very concerned	373	8.3 (6.5)	371	5.8 (6.0)	391	45.9 (10.2)
somewhat concerned	1548	8.0 (5.8)	1542	5.4 (5.0)	1532	47.0 (8.7)
very concerned	1337	8.6 (6.3)	1332	6.0 (5.4)	1329	47.1 (8.9)

### 3.2. Research question 1—main effect of climate concern

As noted above, due to the small number of participants with complete-case data on all exposures, outcomes and confounders (*n* =~420), we focus here on the results from the multiple imputation analyses. The complete-case analysis results are consistent with these multiple imputation results—albeit with considerably greater error—and are presented in the electronic supplementary material (figures S5 and S6, table S7).

**Figure 2.**
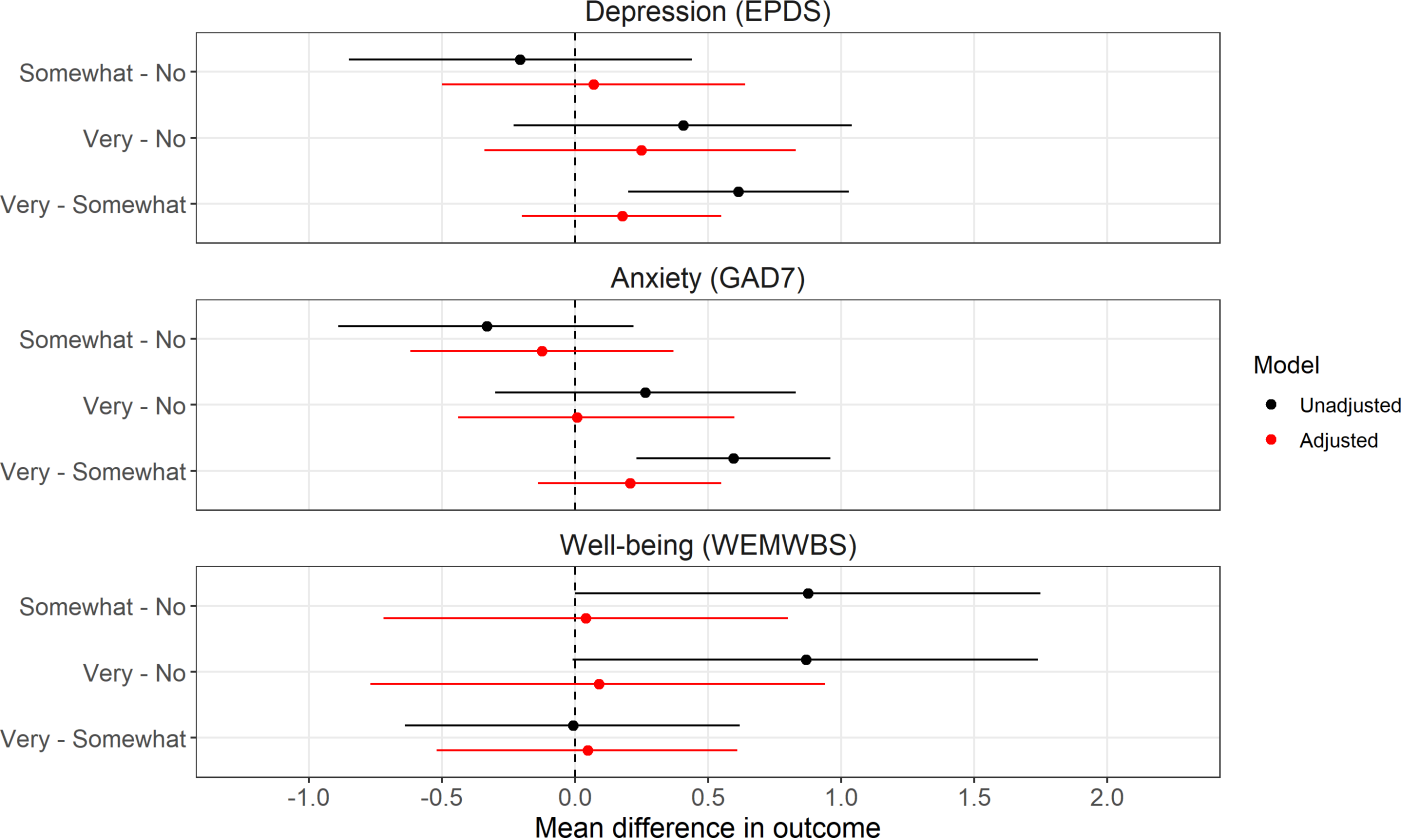
Results from research question 1 on the main effect of climate concern on mental health and well-being from the multiple imputation analyses (*n* = 5146). EPDS = Edinburgh Postnatal Depression Scale; GAD7 = Generalized Anxiety Disorder 7 scale; WEMWBS = Warwick–Edinburgh Mental Well-Being Scale; No = ‘Not at all/Not very concerned’.

Unadjusted results were comparable with those in table 1 above, with depression and anxiety scores higher in ‘very concerned’ participants compared with ‘somewhat concerned’, but with little difference between these two levels and ‘not at all/not very concerned’. Unadjusted well-being scores were marginally higher in both ‘somewhat concerned’ and ‘very concerned’ participants, compared with ‘not at all/not very concerned’. However, after adjustment for the range of plausible confounders, all results were effectively null, indicating little-to-no association between climate concern and any of the mental health or well-being outcomes (figure 2; full numerical results in electronic supplementary material, table S7). As all adjusted results were effectively null, we did not perform sensitivity analyses for unmeasured confounding or report *R^2^* statistics.

### 3.3. Research question 2—effect modification

**Figure 3.**
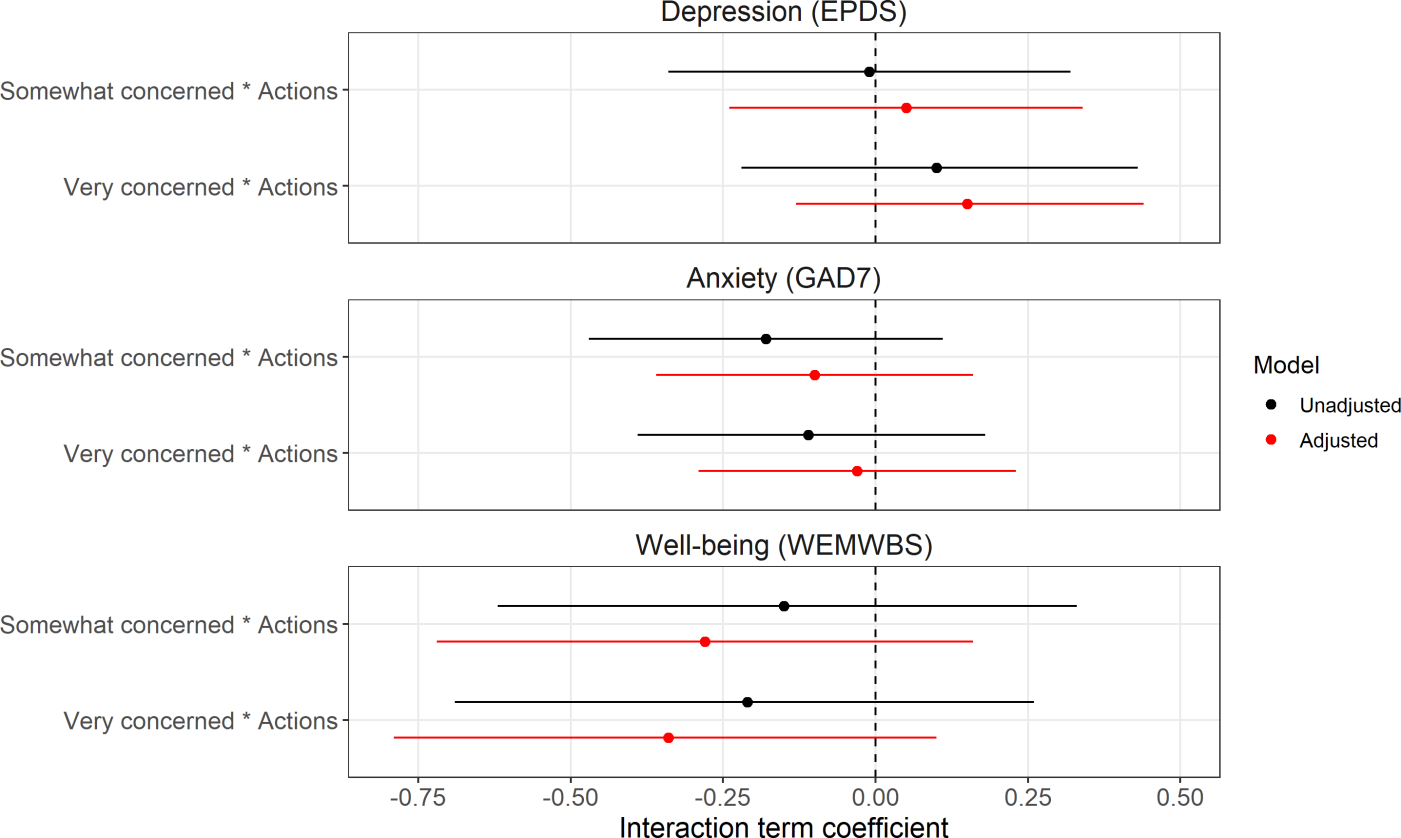
Results from research question 2 regarding potential effect modification of climate concern on mental health and well-being by the total number of individual climate actions performed. Results are from the multiple imputation analyses using the ‘all interactions’ method (*n* = 5146). The reference category for the climate concern interaction term is ‘Not at all/Not very concerned’. EPDS = Edinburgh Postnatal Depression Scale; GAD7 = Generalized Anxiety Disorder 7 scale; WEMWBS = Warwick–Edinburgh Mental Well-Being Scale.

**Figure 4.**
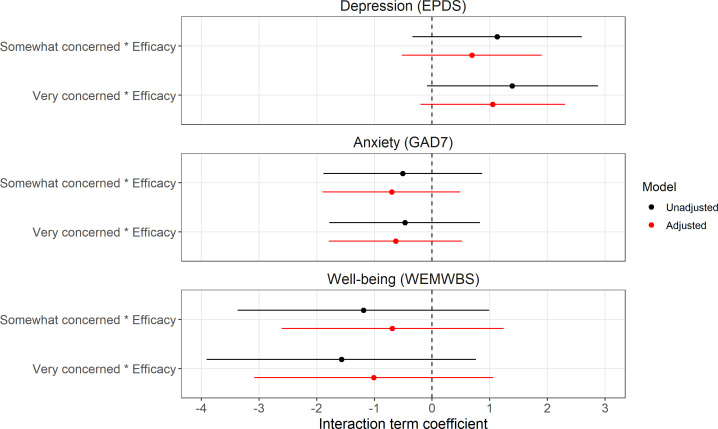
Results from research question 2 regarding potential effect modification of climate concern on mental health and well-being by belief in the efficacy of individual actions to reduce the impact of climate change. Results are from the multiple imputation analyses using the ‘all interactions’ method (*n* = 5146). The reference category for the climate concern interaction term is ‘Not at all/Not very concerned’. EPDS = Edinburgh Postnatal Depression Scale; GAD7 = Generalized Anxiety Disorder 7 scale; WEMWBS = Warwick–Edinburgh Mental Well-Being Scale.

We also found little evidence for effect modification of climate concern on mental health or well-being by either engagement in individual climate actions or belief in the efficacy of climate actions (see figures 3 and 4 for results from the ‘all interactions’ multiple imputation analyses; SMCFCS results were comparable, while complete-case results were also consistent with these imputed results, albeit with much greater uncertainty; see electronic supplementary material, figures S7–S10, with full results in electronic supplementary material, tables S8 and S9).

To help understand the results of these interaction models, predicted mental health and well-being values based on the adjusted models from the ‘all interactions’ and SMCFCS imputation methods are presented in electronic supplementary material, table S10 (for ‘individual climate actions’) and S11 (for ‘belief in efficacy of climate actions’). These show that any effect modification is weak and inconsistent. For instance, predicted values of depression scores are similar regardless of the number of climate actions performed for both ‘not at all/not very concerned’ and ‘somewhat concerned’ levels; while for ‘very concerned’ a greater number of climate actions is associated with somewhat higher depression scores (0 actions = 7.47 (95% confidence interval (CI) = 6.39 to 8.55); 15 actions = 9.65 (95% CI = 8.61 to 10.70)). For anxiety, across all levels of climate concern there was a small positive relationship between the number of climate actions and higher anxiety scores, with little difference between the climate concern categories; while for well-being there was a small positive association between climate actions and greater well-being for all levels of climate concern, but this was marginally stronger among the ‘not at all/not very concerned’ group. Based on these predicted values there was also little evidence for effect modification by belief in the efficacy of climate actions for anxiety or well-being, although belief in the efficacy of climate actions was associated with slightly lower depression scores for the ‘not at all/not very concerned’ level (No/Not sure = 8.53 (95% CI = 7.54 to 9.51); Yes = 7.63 (95% CI = 6.44 to 8.82)), but not for ‘somewhat concerned’ (No/Not sure = 8.48 (95% CI = 7.64 to 9.32); Yes = 8.27 (95% CI = 7.44 to 9.11)) or ‘very concerned’ (No/Not sure = 8.45 (95% CI = 7.53 to 9.37); Yes = 8.61 (95% CI = 7.79 to 9.44)). However, as all interaction terms were weak and had 95% CIs which substantially crossed the null, any evidence for effect modification should be considered weak, at best.

The main effect of climate concern from these interaction imputation models was comparable with the imputation model from research question 1, suggesting that the imputation models with interaction terms were not biased (electronic supplementary material, figures S11 and S12, table S12).

## 4. Discussion

In this population of young adults from the UK we found little evidence for a relationship between climate concern and subsequent mental health and well-being. To the extent these results are causal (which we discuss in more detail below), these results suggest that the average causal effect of climate concern may be close to zero. Although containing many uncertainties—and may be conservative given our use of multiple imputation to boost sample size—our power analyses suggested that we had sufficient power to detect effect sizes of 0.25 standard deviation units or larger (approximately 1.5, 1.3 and 2.25 units for depression, anxiety and well-being, respectively); we cannot therefore rule out the presence of true effect sizes smaller than these values, although whether such small effect sizes are of practical or clinical significance may be questionable. We also found little consistent or convincing evidence that these effects were moderated by either engagement in individual climate actions or belief in the efficacy of climate actions to mitigate the impact of climate change.

Many previous studies have reported a relationship between climate anxiety and worse mental health [3,5,12–15], so why might our results differ? There are many possible explanations. First, the majority of previous studies were cross-sectional and therefore did not control for prior mental health, which could cause both climate anxiety/concern and later mental health and hence confound any causal interpretation (figure 1). While possible, even in our unadjusted analyses—which did not control for prior mental health—there was only a weak relationship between climate concern and mental health and well-being, with ‘very concerned’ participants displaying more depression and anxiety symptoms than ‘somewhat concerned’ participants (figure 2). However, effect sizes were small (approx. 0.6 unit difference, approx. 0.1 of a standard deviation), there were no differences in depression or anxiety compared with ‘not at all/not very concerned’, and ‘somewhat concerned’ and ‘very concerned’ appeared to have marginally *better* well-being than ‘not at all/not very concerned’. Alternative explanations for why the present results differ from previous studies are therefore needed.

One alternative explanation is due to differences in measurement, in that the present study focused on ‘climate concern’, while previous studies largely focused on ‘climate anxiety’. As discussed above, while they are conceptually related and empirically correlated, they are not synonymous [16,23,35], with climate concern representing the less-extreme manifestations on the climate anxiety spectrum. Of note, a recent cross-national study of 11 European countries (published after our Stage I in principle acceptance) explored the relationship between climate worry—conceptually very similar to our measure of ‘climate concern’—and mental health, using longitudinal data from two waves of data collection [58]. Overall, this study found a small positive relationship between climate worry and anxiety, but little relationship with either depression or sleep disturbance. However, there was considerable cross-national variation in these relationships, notably finding little evidence for an association in the UK, therefore partially replicating our results. Perhaps similar longitudinal designs which had measures of climate anxiety (e.g. [12]) may have found stronger evidence for a relationship with subsequent mental health; this is a clear and important open question for future research to explore. Although we cannot extrapolate our results to climate anxiety, our results nonetheless suggest that, on average, there appears to be little/no causal effect of climate *concern* on subsequent mental health or well-being, at least in this UK population of young adults.

While our results indicate a lack of an *average* causal effect of climate concern in this population, there is the possibility of effect heterogeneity; that is, in certain groups of the population climate concern may impact subsequent mental health while in others it may not. For instance, the effect of climate concern on subsequent mental health could perhaps vary by age, gender, prior mental health or being directly affected by climate change, among numerous other factors. Our results do, however, suggest little-to-no effect modification by engagement in individual climate actions or belief in the efficacy of climate actions to impact climate change. These results are somewhat counter to previous research, which has found that engagement in *collective* climate action moderated the negative effects of climate anxiety [17]. This raises the possibility that the collective or social nature of climate action—i.e. being part of a cause with like-minded individuals, along with the social support and interaction this entails—may be a stronger moderator than simply engaging in climate actions *individually*. While beyond the remit of the present study, a more detailed exploration of potential effect modifiers of this relationship may be an interesting avenue for future research. Relatedly, any putative causal effect may also differ between populations [58], as we discuss in more detail below.

### 4.1. Strengths and limitations

Our study possesses many key strengths, including: (i) the use of longitudinal data with baseline mental health data, thus lowering the risk of reverse causality between the exposure and outcome; (ii) a wide range of baseline confounders to minimize the risk of unmeasured confounding biasing results; (iii) the use of a large-scale population-based study to improve both statistical power and generalizability to the target population; and (iv) a range of sensitivity analyses, including multiple imputation for missing data and proposed analyses for unmeasured confounding (even if the latter were not implemented, due to the largely null results). Together, these make the case for a causal interpretation stronger.

However, there are a number of limitations which may threaten both a causal interpretation and the extent to which results are generalizable. We will now briefly discuss the three main threats to causal inference—confounding, selection bias and measurement error [19]—and whether we believe they may cause bias in our analyses. We note here that these are just our assumptions, and it is possible that they may be incorrect; further work will be required to explore this in more depth and see whether our results replicate. We will also end with a brief discussion on generalizability.

*Confounding*. Confounding bias has been discussed in detail above, and we believe that inclusion of the wide range of baseline confounders detailed above (electronic supplementary material, table S3) is sufficient to reduce the risk of confounding bias, particularly the inclusion of baseline mental health and well-being variables. While it is of course possible that other unmeasured confounders which we have not considered may bias these associations, mental health/well-being and sociodemographic factors are known to have strong associations with both climate anxiety and mental health [3] and so are key confounders to adjust for which will hopefully minimize the possibility of unmeasured confounding. As ALSPAC has currently only asked the climate questions once, we were not able to adjust for prior climate concern, which could perhaps be a relevant confounder if it impacts both climate concern at age 30 and subsequent mental health (independent of prior mental health [20]). Having prior measures of climate concern would also have allowed us to strengthen our causal interpretations by assessing whether a *change* in climate concern caused a *change* in mental health/well-being. A related source of confounding bias is due to ‘residual confounding’; that is, if the confounders are measured with error, then their inclusion as covariates may not be sufficient to fully remove confounding bias [19,59]. While possible, many of the prior mental health confounders are based on validated scales, while the inclusion of a wide-range of socioeconomic confounders should increase the probability of capturing socioeconomic position accurately, hopefully reducing the risk of residual confounding. Even if unmeasured or residual confounding is a possibility, the use of sensitivity analyses for unmeasured confounding would have allowed us to explore the levels of unmeasured confounding necessary to alter our interpretations and whether these are plausible or not (again, even though in practice we did not use this approach as the results were largely null).

*Selection bias*. This bias has been discussed above in the ‘missing data’ section, which we will expand upon here. One main concern is that that our imputation procedure was only to approximately 5000 participants in the analytic sample with exposure and/or outcome data, which is around one-third of the full ALSPAC sample size. While this selected sample could theoretically result in bias, we have made this decision for both practical and theoretical reasons. From a practical perspective, as only approximately 30% of the ALSPAC sample have exposure or outcome data, there is a greater chance of model misspecification and resulting error in the imputation model when imputing this large a proportion of missing data for the full approximately 15 000 sample, especially given the lack of valid auxiliary variables to help predict this missing data [60]. From a theoretical perspective, as discussed above the inclusion of factors known to relate to continued ALSPAC participation—such as maternal age, socioeconomic position, prior mental health and offspring sex—in our substantive analysis model is likely to reduce the risk of selection bias by making the ‘missing-at-random’ assumption more plausible. While it is possible that the exposure and outcome may cause selection directly—which would not be corrected by the covariate adjustment method described above—we believe that this is unlikely to result in substantial bias as we feel that participation in ALSPAC is unlikely to be strongly influenced by the exposure climate concern, independent of the other covariates included in our model. This is because ALSPAC is predominantly a health study, with the climate questions embedded within a larger questionnaire, meaning that completion is unlikely to be strongly associated with climate awareness and concerns. While this is an assumption, if the exposure has little relation to selection then collider stratification selection bias is unlikely to strongly bias results [50], although we cannot rule out effect modification selection bias if variables which moderate the exposure–outcome relationship themselves cause selection [22].

*Measurement error*. As our outcomes have been assessed using well-validated scales, we anticipate little measurement error in our outcomes, minimizing the risk of bias. Prior ALSPAC research has shown that self-reported responses to potentially sensitive topics, such as mental health and medical records, are comparable with ‘gold-standard’ measures [61], providing some assurance against bias due to measurement error. However, as discussed above it is possible that our measure of climate concern may not fully capture all relevant aspects of climate anxiety; that is, our exposure may be measured with error if climate concern is intended as a proxy for climate anxiety. Although climate concern is certainly an important aspect of climate anxiety, and previous studies have shown that climate concern and climate anxiety are related and may measure similar constructs [16,23,35], this study likely overlooks many of the specific thoughts and behaviours related to climate anxiety which may be captured by more detailed and validated scales (e.g. [12]). As noted above, while this is to some extent unavoidable given our use of secondary ALSPAC data, it is an important limitation to consider. For instance, the focus on climate concern could perhaps result in an underestimate of the true effect size of the impact of climate anxiety on mental health, as only those suffering from severe climate anxiety—and not merely those very concerned about climate change—may have worse subsequent mental health. We hope that future research can combine the strengths of our study—large-scale broadly-representative longitudinal data—with well-validated measures of climate anxiety to explore if/how they differ from our results using just climate concern. We also note that our ‘individual climate actions’ effect modifier could be measured with error as the question asked whether participants had performed any of these actions, regardless of frequency (electronic supplementary material, table S1). This could lead to a dilution of any potential effect modification if, for instance, engaging repeatedly in these climate actions moderated the relationship between climate concern and mental health/well-being more compared with only performing these actions once; yet in these analyses both situations are impossible to separate and would be grouped together.

*Generalizability:* As this sample is based on ALSPAC offspring born in the early 1990's in the Bristol/Avon area of south-west England, the extent to which results may be generalizable to the wider UK population—or beyond—is unclear. For instance, ALSPAC offspring are more ethnically homogenous compared with the wider UK population (approx. 4% of ALSPAC offspring have an ethnicity other than White versus approx. 14% in the wider UK population) and are less likely to come from low income households [26,27]. The extent to which results would generalize to ages beyond those studied here (early 30's) is also difficult to predict, particularly to younger ages who may be more affected by climate anxiety [2,8]. Finally, we note that the city of Bristol is a very ‘green’ city, being the first in the UK to declare a Climate and Ecological Emergency [62] and one of the first to elect a Green Party member of parliament. It is possible that this could alter the relationship between climate concern and mental health, compared with other less ‘green’ areas; for instance, those concerned about climate change might have a larger social support network of like-minded individuals, potentially mitigating any impacts on mental health or well-being. However, as noted above, a recent paper using similar methods and measures also found little evidence for a relationship between climate worry and mental health in a UK sample [58], suggesting that these results may be somewhat generalizable to the wider UK population. This study also found considerable variation in this relationship by country; replicating and extending these cross-cultural results, and understanding the reasons for such cross-cultural variation—e.g. role of government (in)action regarding climate policies, direct exposure to climate related events, etc.—is therefore a key area for future research.

## 5. Conclusion

In summary, our findings suggest that, given our causal assumptions, in this UK population of young adults there is little evidence for an average causal effect of climate concern on mental health and well-being. We also find little evidence for effect modification by engagement in individual climate actions or belief in the efficacy of climate actions to mitigate the impact of climate change. By using data from a large-scale longitudinal population-based study, our study provides stronger evidence for a causal interpretation compared with many previous studies, which have largely been cross-sectional. However, many open questions still remain, such as the extent to which these results regarding climate concern extend to climate anxiety, whether other factors moderate this relationship, and whether these results are generalizable to other populations. We hope to see other research in this area making use of similar longitudinal datasets to better understand the impact of climate change on mental health.

## Data Availability

ALSPAC data access is through a system of managed open access. Information about access to ALSPAC data is given on the ALSPAC website (http://www.bristol.ac.uk/alspac/researchers/access/) and in the ALSPAC data management plan (ALSPAC_Access_Policy.pdf). Data used for this submission will be made available on request to the Executive (alspac-exec@bristol.ac.uk). The datasets presented in this article are linked to ALSPAC project number B4572, please quote this project number during your application. Analysis code and synthetic ALSPAC datasets (created using the ‘synthpop’ R package [63]) are openly available on D.M.-S.’s GitHub page [64]. As raw ALSPAC data cannot be released, these synthesized datasets are modelled on the original data, thus maintaining variable distributions and relations among variables (albeit not perfectly), while at the same time preserving participant anonymity and confidentiality, thus allowing this research to be ‘quasi-reproducible’ [65]. Please note that while these synthetic datasets can be used to follow the analysis scripts, as data are simulated they should not be used for research purposes; only the actual, observed, ALSPAC data should be used for formal research and analyses reported in published work. For the Stage I analysis plan, scripts demonstrating the proposed analyses using simulated data, and for the power analyses, can be found on OSF [57] (these scripts are also available in the above GitHub repository). Supplementary information is available online at [66], as part of the wider OSF project page [57].
